# Growth hormone secretagogue receptor deficiency in mice protects against obesity‐induced hypertension

**DOI:** 10.1002/phy2.240

**Published:** 2014-03-20

**Authors:** Louise E. Harris, David G. Morgan, Nina Balthasar

**Affiliations:** ^1^ School of Physiology and Pharmacology University of Bristol Bristol BS8 1TD UK; ^2^ AstraZeneca R&D Mereside Alderley Park SK10 4TG UK; ^3^ Present address: School of Pharmacy Keele University Keele ST5 5BG UK

**Keywords:** Diet‐induced obesity, ghrelin, growth hormone secretagogue receptor, hypertension

## Abstract

Growth hormone secretagogue receptor (GHS‐R) signaling has been associated with growth hormone release, increases in food intake and pleiotropic cardiovascular effects. Recent data demonstrated that acute GHS‐R antagonism leads to increases in mean arterial pressure mediated by the sympathetic nervous system in rats; a highly undesirable effect if GHS‐R antagonism was to be used as a therapeutic approach to reducing food intake in an already obese, hypertensive patient population. However, our data in conscious, freely moving GHS‐R deficient mice demonstrate that chronic absence of GHS‐R signaling is protective against obesity‐induced hypertension. GHS‐R deficiency leads to reduced systolic blood pressure variability (SBPV); in response to acute high‐fat diet (HFD)‐feeding, increases in the sympathetic control of SBPV are suppressed in GHS‐R KO mice. Our data further suggest that GHS‐R signaling dampens the immediate HFD‐mediated increase in spontaneous baroreflex sensitivity. In diet‐induced obesity, absence of GHS‐R signaling leads to reductions in obesity‐mediated hypertension and tachycardia. Collectively, our findings thus suggest that chronic blockade of GHS‐R signaling may not result in adverse cardiovascular effects in obesity.

## Introduction

Ghrelin is an acylated peptide hormone, mainly secreted from the gastrointestinal mucosa and it functions as the endogenous ligand to the growth hormone secretagogue receptor 1a (GHS‐R) (Kojima et al. 1999). Although originally identified as a potent stimulator of pituitary growth hormone secretion and orexigenic hormone [for review: (Van Der Lely et al. 2004)], more recently ghrelin's roles have extended to include food‐reward behaviors and a host of cardiovascular actions (Perello et al. 2010; Granata et al. 2011).

GHS‐Rs are expressed in various CNS areas and peripheral tissues. Key CNS sites include nuclei of the mediobasal hypothalamus, ventral tegmental area of the midbrain and hindbrain sites, including the nucleus of the solitary tract (NTS), dorsal motor nucleus of the vagus nerve (DMV) and area postrema (Zigman et al. 2006). GHS‐R expression has also been reported in heart and blood vessels, although the latter may be area‐ and species‐specific (Howard et al. 1996; Gnanapavan et al. 2002; Callaghan et al. 2012).

In addition to inotropic and cardioprotective effects against ischemia, ghrelin and its mimetics have been found to exert significant vasodilatory action [for review see (Granata et al. 2011)]. Studies in humans found that intravenous (i.v.) injection of ghrelin elicits a decrease in blood pressure (Nagaya et al. 2001; Lambert et al. 2011), although these effects of ghrelin and its mimetics are not always consistent (Bisi et al. 1999). In rabbits, i.v. injection of ghrelin caused dose‐related decreases in mean arterial pressure (MAP) without significant changes in renal sympathetic nerve activity (RSNA) (Matsumura et al. 2002). The hypotensive action of ghrelin was also confirmed in rats (Li et al. 2009). Additionally, intracerebroventricular (i.c.v.) injection of ghrelin caused a significant decrease in MAP, HR, and RSNA, suggesting that systemically administered ghrelin acts, at least in part, via the CNS (Matsumura et al. 2002). Indeed, NTS injections of ghrelin significantly decrease MAP and HR by suppression of sympathetic nerve activity in rats (Lin et al. 2004). Longer‐term (10‐day) i.c.v. infusion of ghrelin in rats also cause mild reductions in MAP and HR by reducing sympathetic tone to the heart, whereas i.v. infusions had no such effect (Freeman et al. 2013). Furthermore, studies with the synthetic GHS‐R antagonist [d‐Lys‐3]‐GHRP‐6 showed a significant sympathetic nervous system‐mediated, dose‐dependent increase in both HR and MAP in conscious rats (Vlasova et al. 2009).

GHS‐R antagonism may be an attractive therapeutic approach to reducing food intake, however, concomitant increases in blood pressure in an already obese and most likely hypertensive patient population is highly undesirable. Given discrepancies in the literature on ghrelin's cardiovascular actions, in combination with absence of mechanistic insight into ghrelin's cardiovascular effects, we sought to investigate long‐term cardiovascular effects in the absence of GHS‐R signaling.

Obesity is a strong, independent risk factor for the development of cardiovascular disease; indeed 75% of hypertensive disease is related directly to obesity (Go et al. 2013). Hypertension itself is a highly significant risk factor for the development of all manifestations of cardiovascular disease, including coronary heart disease, stroke and heart failure. The etiology of obesity‐induced hypertension includes insulin‐ and leptin‐mediated increases in sympathetic nervous system activity as well as an increased renin‐angiotensin‐aldosterone system activity (Hall et al. 2010). Given ghrelin's autonomic modulation of the cardiovascular system, we sought to investigate GHS‐R‐mediated roles in obesity‐induced hypertension.

Of note, ghrelin‐ and GHS‐R‐mediated cardiovascular effects in rodents have to date been solely investigated using pharmacological approaches mostly in anaesthetized animals. In addition, room for a discussion around alternative ghrelin receptors remains (Seim et al. 2011).

Here, we investigate the cardiovascular effects of chronic absence of GHS‐R signaling in freely moving, conscious, lean and diet‐induced obese GHS‐R‐deficient mice.

## Materials and Methods

### Animals

Studies were performed in accordance with the UK Animals (Scientific Procedures) Act 1986 and with approval of the University of Bristol Ethical Review Group.

Wild‐type (WT) and GHS‐R null littermate mice on a C57BL/6J background were obtained from AstraZeneca (GHS‐R1A KO mice originally obtained from Deltagen (San Carlos, CA, USA); colony maintained from heterozygous breeding pairs) (Egecioglu et al. 2010). Genotyping was performed at weaning by ear notch PCR, using the following primer sequences in 5′–3′: GCTACTTCGCCATCTGCTTC (upstream‐targeted exon), AAGACGCTCGACACCCATAC (within‐targeted exon) and GGGTGGGATTAGATAAATGCCTGCTCT (within‐targeting cassette). On arrival at the University of Bristol, all mice were single‐caged with environmental enrichment (nesting and cardboard tubes). All mice were housed under controlled conditions of temperature (21–22°C), humidity (50%), and 12:12 h light/dark cycle. Mice were placed on a standard chow diet (2016 Teklad Global 16% Protein Diet; Harlan, Shardlow, UK) and had free access to tap water.

### Telemetry

Male GHS‐R knockout and wild‐type littermate mice received radio‐telemeter implants (TAIIPA‐C10; Data Sciences International, St Paul, MN, USA) in the carotid artery, as described earlier (Kaidi et al. 2007). In brief, mice were anaesthetized with 3,3,3‐Tribromoethanol (250 mg/kg; Sigma, Gillingham, UK) and a small incision was made from the ventral neck. The right common carotid artery was isolated, ligated, and cannulated using the catheter of the pressure transducer, and further secured using nonabsorbable sutures (Look 5.0; Harvard Apparatus, Edenbridge, UK). The body of the transmitter was placed in a subcutaneous pocket down the left flank of the mouse. Mice continued to be singly caged after surgery with environmental enrichment. Freely moving mouse cardiovascular parameters were monitored on individual receiver pads after a minimum recovery period of 10 days post‐surgery, typically for 5 min every hour for 48‐h (1000 Hz sampling frequency), using the telemetry data acquisitions system (Dataquest A.R.T.™ 4.0; Data Sciences International). Measurements were split into light (9 am–5 pm), dark (9 pm–5 am) phase, and average 24 h.

### Acute HFD‐feeding

Mice (7 month) with radio‐telemeter implants were placed on a high‐fat diet (Research Diets D12331, 58 kcal% fat w/sucrose Surwit Diet, New Brunswick, NJ) for 12 days, during which food intake, body weight and cardiovascular parameters were recorded every other day. Cardiovascular parameters were recorded during the dark phase (9 pm–5 am) for 5 min every hour.

### Diet‐induced obesity

Mice (7 month) were placed on a high‐fat diet (Research Diets D12331, see above) for 15 weeks prior to telemetry surgery and weighed weekly. After assessment of fasting glucose levels (16 h over‐night fast) by tail tip and hand‐held glucometer, mice underwent telemetry surgery (as described above). Cardiovascular parameters were recorded over a 48‐h period, recording for 5 min every hour. Measurements were split into light (9 am–5 pm), dark (9 pm–5 am) phase, and average 24 h.

### DSI and statistical analysis

Cardiovascular parameters collected by Dataquest A.R.T 4.0 (Data Sciences International) include mean arterial pressure (MAP), systolic and diastolic blood pressure (SBP, DBP), interbeat intervals (IBI) and dPdt (cardiac contractility).

Power spectral analysis to obtain frequency data for systolic blood pressure variability (SBPV) analysis were performed after interpolating SBP and IBI (50 Hz, 2nd order), detrending and mean suppressing raw data. The power spectra were divided into two domains: high‐frequency (HF) 1.5–4 Hz and low‐frequency (LF) 0.15–1.5 Hz (Baudrie et al. 2007) and data were normalized into a Hanning distribution, with one overlapping.

To assess spontaneous baroreflex sensitivity, mean arterial pressure data were exported into hemolab baroreflex analysis software (http://www.haraldstauss.com/HemoLab/HemoLab.php). The hemolab analyzer identifies rises and falls in MAP and HR, which occur in a linear fashion and involve three or more consecutive points; a method previously validated (Moffitt et al. 2005; Stauss et al. 2006).

Group data are expressed, as mean ± SEM. Data were compared using Student's *t*‐tests, one‐ or two‐way repeated measures analyses of variance (ANOVA) followed by Bonferroni's procedure for multiple comparisons, where appropriate using GraphPad Prism software (GraphPad Software, Inc., La Jolla, CA).

### CORT measurements

Male 5 months‐old WT and GHS‐R mice were singly caged, acclimatized to handling for 3 weeks and on the day of experiment tail pricked for 10 μL blood collection in heparinized capillary tubes every 2 h from 8 am until 22 pm 1:5 dilutions of serum samples were assayed using a Corticosterone HS EIA kit (Immunodiagnostic Systems Limited, Boldon, UK) according to manufacturer's instruction.

## Results

### GHS‐R deficiency leads to reduced systolic blood pressure variability

**Figure 1:**
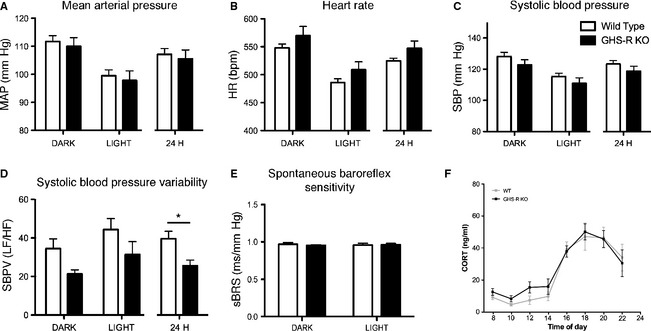
Growth hormone secretagogue receptor (GHS‐R) is critical for appropriate regulation of systolic blood pressure variability. Radio‐telemetry measurements in ad libitum chow fed, adult male wild‐type (WT, open bars) and GHS‐R KO (black bars) littermate mice. (A) Mean arterial pressure. (B) Heart rate. (C) Systolic blood pressure. (D) Spectral analysis of systolic blood pressure variability as LF/HF ratio. GHS‐R signaling is critical in the maintenance of appropriate autonomic control of blood pressure oscillations. (E) Spontaneous baroreflex sensitivity. Dark phase (9 pm–5 am)/light phase (9 am–5 pm) phase: two‐way repeated measures ANOVA for time and genotype, 24 h: *t*‐test; *P < 0.05 for genotype (WT *n* = 6; GHS‐R KO *n* = 8). (F) Diurnal corticosterone (CORT) profiles in WT (gray lines) and GHS‐R KO (black lines) mice (*n* = 8).

Male, 6‐month‐old WT and GHS‐R KO mice had identical body weight (WT = 30.49 ± 0.70 g, GHS‐R KO = 30.58 ± 0.19 g). GHS‐R deficiency had no effect on mean arterial pressure (MAP), heart rate (HR) or systolic blood pressure (SBP) (Fig.**** 1A–C). Diastolic blood pressure and cardiac contractility were also unaffected (data not shown). Circadian rhythmicity of cardiovascular parameters was maintained independent of GHS‐R genotype (Fig. 1A–D) and no differences in diurnal corticosterone variations were noted (Fig. 1F). Identifying the frequency components of blood pressure variability by power spectral analysis can provide important information on blood pressure control mechanisms, which operate at different response times. In terms of autonomic control of blood pressure variability, a low‐frequency zone (LF) has been identified, which reflects sympathetic control, whereas a high‐frequency zone (HF) is under vagal control (Baudrie et al. 2007). Spectral analysis of systolic blood pressure variability showed a significant reduction in the LF/HF power ratio in GHS‐R KO mice, which was most prominent over a 24‐h period (*P* < 0.05, Fig. 1D). Spontaneous baroreflex sensitivity (sBRS) was unaltered by GHS‐R deficiency (Fig. 1E). These data suggest that long‐term GHS‐R deficiency does not lead to overt cardiovascular abnormalities, but modulates autonomic control of blood pressure oscillations.

### GHS‐R deficient mice are resistant to high‐fat diet‐mediated increases in systolic blood pressure variability

**Figure 2:**
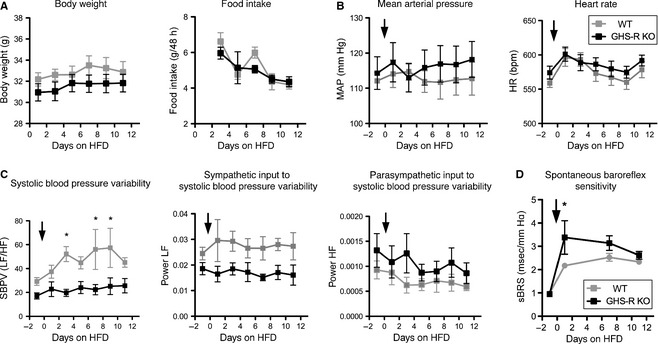
Growth hormone secretagogue receptor (GHS‐R) deficient mice are resistant to acute high‐fat diet (HFD)‐mediated increases in systolic blood pressure variability. Adult male WT (gray lines) and GHS‐R deficient mice (black lines) were fed a HFD for 12 days and dark‐phase cardiovascular parameters recorded every other day. (A) Body weight and food intake over 12‐day period. (B) Mean arterial pressure and heart rate. (C) Spectral analysis of systolic blood pressure variability (LF/HF ratio and HF and LF alone). While in WT mice SBPV increases over time of HFD‐feeding, this response is absent in GHS‐R KO, due, in most part, to a lack of increase in sympathetic control of blood pressure oscillations. (D) Spontaneous baroreflex sensitivity. sBRS is increased in both WT and GHS‐R KO mice in response to HFD‐feeding, however, this response appears increased in GHS‐R KO mice. Arrow indicates start of HFD‐feeding. (two‐way ANOVA repeated measures for time and genotype, *P < 0.05 for genotype, WT *n* = 6; GHS‐R KO *n* = 7).

During 12 days of high‐fat diet (HFD) feeding no difference in body weight or food intake was observed between genotypes of adult, 7 month‐old male mice. GHS‐R KO and WT mice both reduced average food intake to account for increased calorie diet (Fig. 2A). Cardiovascular parameters were recorded during the dark phase every other day for 12 days. MAP remained unaltered in response to acute HFD‐feeding in both WT and GHS‐R KO mice. Although HR was slightly elevated in response to HFD‐feeding, this response was identical in WT and GHS‐R KO mice (Fig. 2B).

As described above, SBPV was significantly decreased in GHS‐R KO mice during chow diet‐feeding (Fig. 1D); this phenomenon was exacerbated during acute HFD‐feeding. While in WT mice, SBPV increased over time of HFD‐feeding, this response was absent in GHS‐R KO mice (*P* < 0.05, Fig. 2C), along with a lack of increase in sympathetic control of blood pressure oscillations (LF power, Fig. 2C). sBRS was increased in both WT and GHS‐R KO mice in response to HFD‐feeding (Fig. 2D), however the initial, immediate response was significantly stronger in GHS‐R KO mice (*P* < 0.05).

### Diet‐induced obese GHS‐R deficient mice have lower MAP than their WT littermates despite similar body weights

**Figure 3:**
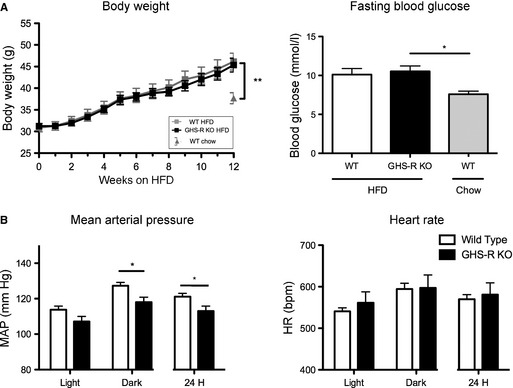
Diet‐induced obese (DIO) Growth hormone secretagogue receptor (GHS‐R) deficient mice have lower MAP than their WT littermates despite similar body weights. Male adult WT and GHS‐R KO mice were fed a HFD for 12 weeks and cardiovascular parameters recorded every other day. (A) HFD‐fed WT and GHS‐R KO mice significantly increase in body weight and show fasting hyperglycemia (one‐way ANOVA, *P < 0.05, **P < 0.01, WT chow *n* = 9, WT HFD *n* = 10, GHS‐R KO *n* = 9). (B) Radio‐telemetry measurements in DIO WT and GHS‐R KO littermate mice after 12 weeks of HFD feeding. GHS‐R KO mice have lower MAP than WT littermates despite similar weight gain on HFD. (dark phase (9 pm–5 am)/light phase (9 am–5 pm) two‐way repeated measures ANOVA for time and genotype; 24 h *t*‐test; *P < 0.05 for genotype; WT *n* = 8; GHS‐R KO *n* = 6).

During longer‐term HFD feeding adult, male mice were weighed every week for 12 weeks prior to surgical implantation of radio‐transmitters. Body weight of GHS‐R KO and WT littermates significantly increased by 47.5% (approximately 15 g, *P* < 0.01) over the 12‐week period with no difference in body weight gain between genotypes (Fig. 3A). HFD‐fed GHS‐R KO and WT mice had diet‐induced obese (DIO) hallmark fasting hyperglycemia compared to WT age‐matched control chow‐fed mice (*P* < 0.05, Fig. 3A).

Cardiovascular physiology of these obese WT and GHS‐R KO mice was monitored for a 48‐h period. While HR remained unchanged between genotypes, GHS‐R KO mice had significantly decreased MAP compared to WT littermates, evident particularly during the dark phase (*P* < 0.05, Fig. 3B). These data suggest that absence of ghrelin signaling protects mice from developing DIO‐mediated hypertension despite significant HFD‐mediated weight gain.

### GHS‐R deficient mice are protected from HFD‐induced hypertension

**Figure 4:**
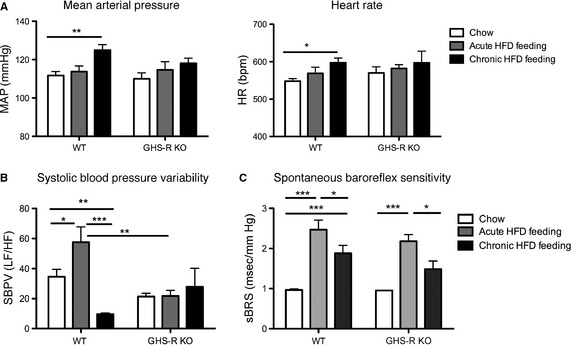
Growth hormone secretagogue receptor (GHS‐R) deficient mice are protected from HFD‐induced hypertension. Comparison of dark‐phase cardiovascular phenotypes in mice fed chow (white bars), HFD for 12 days (gray bars) or 12 weeks (black bars). (A) Mean arterial pressure and heart rate. GHS‐R deficient mice are protected against DIO‐mediated hypertension and increases in HR. (B) Power spectral analysis of SBPV. SBPV responses to diet are absent in GHS‐R KO mice. (C) Spontaneous baroreflex sensitivity. (two‐way ANOVA for diet and genotype; *P < 0.05, **P < 0.01, ***P < 0.001, WT *n* = 6–8, KO = 5–8).

To elucidate the cardiovascular physiological differences between chow, acute and long‐term HFD‐fed mice, average dark‐phase measurements were compared between chow, day‐12 acute HFD, and chronic HFD‐fed mice. Obese chronic HFD‐fed WT mice had significantly increased MAP and HR from chow levels (*P* < 0.01 and *P* < 0.05, respectively, Fig. 4A). However, similarly obese HFD‐fed GHS‐R KO mice had no significant alterations in MAP or HR from chow levels and were thus protected from obesity‐induced hypertension and tachycardia.

Interestingly, in WT mice SBPV increased with acute HFD‐feeding (*P* < 0.05), but was significantly blunted in chronic HFD‐feeding (*P* < 0.01 compared to chow and *P* < 0.001 compared to acute HFD, Fig. 4B). However, in GHS‐R deficiency no alteration of SBPV was observed in response to HFD‐feeding. sBRS was comparable between WT and GHS‐R KO littermates with increases in response to acute HFD‐feeding (initially exaggerated in GHS‐R KO mice, both genotypes *P* < 0.001 compared to chow) which were reduced in chronic HFD‐feeding (both genotypes *P* < 0.01 compared to acute HFD‐feeding, Fig. 4C).

## Discussion

Combining radio‐telemetry techniques with genetically modified mouse models, allows the assessment of the roles of individual genes in long‐term regulation of cardiovascular parameters in freely moving conscious animals. In contrast to previous studies investigating cardiovascular pharmacological GHS‐R activity modulation, data here represent the cardiovascular phenotypes resulting from long‐term GHS‐R signaling deficiency. However, as GHS‐R KO mice used in these studies lack GHS‐Rs throughout development and life, mechanisms that may compensate for the loss of GHS‐R signaling may mask some effects that can be seen with acute GHS‐R inhibition. Ultimately, effects described in GHS‐R KO mice are thus physiological roles of GHS‐R signaling that cannot be compensated for. In addition, GHS‐Rs have been described to have high constitutive activity (Mear et al. 2013) and ablation of this ligand‐independent constitutive activity without affecting ghrelin‐mediated GHS‐R signaling has significant consequences for stature and weight alone (Wang et al. 2004). The predictive value of life‐long GHS‐R deletion to chronic pharmacological inhibition thus needs to be viewed with caution.

Consistent with previous reports (Zigman et al. 2005) GHS‐R deficiency did not cause body weight differences in adult male mice; importantly, cardiovascular effects in GHS‐R deficiency are thus not secondary to body weight changes. However, as these are global KO mice and the GHS‐R is expressed both centrally and peripherally, it is not possible to determine the exact tissue origin of effects.

In brief, our data suggest a significant role for deficiency in GHS‐R signaling in the protection against diet‐induced obesity‐mediated hypertension, despite considerable diet‐mediated weight gain. Protection against diet‐mediated hypertension may be downstream of favorable changes in autonomic control mechanisms of blood pressure variability, including suppression of sympathetic dominance.

### GHS‐R signaling is involved in the maintenance of systolic blood pressure variability

GHS‐R deficiency does not lead to an overt cardiovascular phenotype, however, spectral analysis of SBP showed reduced SBPV in GHS‐R KO mice. Alterations in SBPV in GHS‐R KO mice may be downstream of an altered GH/IGF‐I axis, as reduced IGF‐I levels have been reported in GHS‐R KO mice (Sun et al. 2004). Furthermore, we cannot exclude the possibility of ghrelin effects mediated by receptors other than the GHS‐R; these have certainly been described (Benso et al. 2004). However, as ghrelin levels in GHS‐R KO mice have been reported to be normal (Sun et al. 2004), this is unlikely to have a significant effect on SBPV differences. An altered stress axis is also unlikely to contribute to the SBPV effect as GHSR‐deficient mice have normal diurnal corticosterone rhythms. Acute pharmacological inhibition of the GHS‐R in conscious rats has been shown to lead to increased MAP via sympathetic nervous system activation (Vlasova et al. 2009), highlighting the potential for different physiological responses to acute inhibition versus chronic absence of GHS‐R and perhaps also putative effects of GHS‐R antagonists on other non‐GHS‐R receptors [e.g., (Patel et al. 2012)]. Furthermore, i.c.v. ghrelin infusion over 10 days caused mild reductions in MAP and HR, however no such effect was observed with i.v. infusion, further highlighting ghrelin's differential effects depending on injection route (Freeman et al. 2013). In addition, pharmacological studies using exogenous ghrelin are complicated by its conversion into des‐acyl‐ghrelin as well as by effects on GHS‐R‐mediated growth hormone release, making comparison to data manipulating the endogenous ghrelin system difficult. Global, long‐term deletion of GHS‐R signaling has no effect on MAP or HR; our data thus suggest that longer‐term GHS‐R antagonism, as for example in a food intake reducing therapy, may not result in increased MAP – a strategy that deserves further attention.

### Rapid increase in sympathetic control of SBPV and sBRS in response to HFD feeding, followed by hypertension, tachycardia, and onset of other negative prognostic indicators of cardiovascular disease

Short‐term HFD‐feeding over 12 days caused immediate, significant and expected reductions in food intake to account for increased calorie content of the diet. While there was no effect on MAP over the 12 days of HFD, the immediate increase in heart rate is likely part of a stress response to alteration of the diet; in contrast, chronic HFD‐feeding leads to obesity accompanied by sustained hypertension and tachycardia. Indeed, diet‐induced obesity‐mediated effects on the cardiovascular system are well recognized in humans and rodents alike (Hall et al. 2000; Esler et al. 2006). Spectral analysis of blood pressure oscillations revealed immediate, significant increases in SBPV in response to the diet, driven mainly by an increase in sympathetic control (LF component) with a milder concomitant reduction in parasympathetic influence (HF component). Previous data have suggested that hypertension is associated with sympathetic dominance (Esler et al. 2006); our data show that the increase in sympathetic control of SBPV in response to HFD is immediate. This autonomic imbalance may over time drive the hypertension observed with diet‐induced obesity. Importantly, sBRS is increased immediately in response to HFD‐feeding, likely in a bid to maintain MAP in the face of HFD‐feeding.

In chronic HFD‐feeding, once diet‐induced obesity with concomitant fasting hyperglycemia, hypertension, and tachycardia has set in, SBPV is significantly suppressed. The initial rise in sBRS is also significantly blunted in diet‐induced obesity, indicating perhaps a failure to maintain appropriate blood pressure oscillations and baroreflex control in the face of long‐term high‐fat diet exposure. Reduced sBRS has indeed been correlated with increased insulin‐resistance (Lucini et al. 2006) and diet‐induced obesity in rodents and humans (Dangardt et al. 2011; Latchman et al. 2011; Fardin et al. 2012; McCully et al. 2012).

### GHS‐R deficiency protects from diet‐induced obesity‐mediated hypertension

No significant differences in body weight gain were observed in GHS‐R KO mice and their WT littermates during acute or chronic HFD‐feeding. Previous studies using different strains of GHS‐R KO mice have shown diverse results in terms of body weight responses to HFD‐feeding. Zigman et al. (2005) report that male GHS‐R‐deficient mice have no body weight phenotype on a chow diet, while they are partially protected from diet‐induced obesity when placed on a HFD at 4 weeks of age. On the other hand, Sun et al. (2008) demonstrate that male GHS‐R KO mice are significantly lighter than their WT littermates on chow and HFD, but gain similar amounts of weight during HFD‐feeding from 16 weeks of age; the authors thus argue against GHS‐R deficiency protecting from diet‐induced obesity. Different animal models, background strains, diet compositions and ages of mice may be to blame for discrepancies between these and our own studies. In our hands, the equal body weight gain and development of fasting hyperglycemia in HFD‐fed WT and GHS‐R KO mice allows direct comparison of cardiovascular parameters without metabolic confounds. Despite significant weight gain, GHS‐R deficiency protects mice from HFD‐induced hypertension and tachycardia. Thus, in contrast to acute, pharmacological GHS‐R antagonist studies in conscious rats reporting increases in MAP (Vlasova et al. 2009), we find protective effects of chronic GHS‐R deficiency against obesity‐induced hypertension. Thus, while few and only modest GHS‐R antagonist effects on body weight and food intake have been described (Xin et al. 2006; Esler et al. 2007; Rudolph et al. 2007; Puleo et al. 2012), GHS‐R antagonism may provide beneficial cardiovascular effects in obesity.

Our data further suggest that GHS‐R signaling is critically involved in the regulation of SBPV in response to HFD feeding, with a shift away from sympathetic dominance in the immediate response to HFD‐feeding of GHS‐R KO mice. The immediate sBRS response to HFD‐feeding is exaggerated in GHS‐R KO mice, indicating perhaps that peripheral (e.g., blood vessel) GHS‐R signaling may normally act to diminish the baroreflex response, due to its vasodilatory effects.

Overall, our data demonstrate a significant role for GHS‐R signaling in the autonomic modulation of blood pressure oscillations, possibly by triggering baroreflex‐mediated sympathoexcitation in HFD‐feeding. Previous data have shown intravenous ghrelin‐mediated increases in muscle sympathetic nerve activity in both lean and obese humans (Lambert et al. 2011; Krapalis et al. 2012) although i.c.v. and NTS injections of ghrelin significantly suppressed sympathetic nerve activity in rats (Lin et al. 2004; Freeman et al. 2013). Our data support a role for ghrelin‐mediated increases of sympathetic outflow, which may be downstream of peripheral vascular effects, explaining ghrelin's differential autonomic effects depending on injection route.

Importantly, sexual dimorphism in HFD‐mediated body weight gain has been noted in male versus female GHS‐R KO mice, with females being more affected by the diet (Zigman et al. 2005). In future experiments it will be important to explore whether a sexual dimorphism is also apparent in cardiovascular responses to GHS‐R deficiency, as this might have significant implications for potential therapeutic outcomes. Future experiments, using mice with tissue‐/neuronal‐subpopulation‐specific expression of GHS‐R (Zigman et al. 2005) are needed to determine the tissue/CNS site responsible for GHS‐R deficiency's protective effects against the development of hypertension.

## Conclusions

In conclusion, we demonstrate a role for GHS‐R signaling in the autonomic regulation of blood pressure variability in basal states, as well as in response to changes in diet. We show that absence of GHS‐R signaling protects against obesity‐induced hypertension downstream of favorable changes in autonomic control mechanisms, including suppression of sympathetic dominance. Future pharmacological studies will need to identify whether long‐term GHS‐R antagonism may have similar beneficial cardiovascular effects in diet‐induced obesity.

## Conflict of Interest

David Morgan was at the time an employee of the AstraZeneca CVGI Innovative Medicines Unit.
